# Annual Wormwood Leaf Inhibits the Adipogenesis of 3T3-L1 and Obesity in High-Fat Diet-Induced Obese Rats

**DOI:** 10.3390/nu9060554

**Published:** 2017-05-28

**Authors:** Yuno Song, Soo-Jung Lee, Sun-Hee Jang, Tae Hoon Kim, Hong-Duck Kim, Sung-Woo Kim, Chung-Kil Won, Jae-Hyeon Cho

**Affiliations:** 1 Institute of Animal Medicine, College of Veterinary Medicine, Gyeongsang National University, Jinju 660-701, Korea; yunosong0805@gmail.com (Y.S.); sunhee5321@naver.com (S.-H.J.); wonck@gnu.ac.kr (C.-K.W.); 2 Department of Foods and Nutrition, Gyeongsang National University, Jinju 660-701, Korea; bodry96@hanmail.net; 3 Department of Food Science and Biotechnology, Daegu University, Gyungsan 712-714, Korea; skyey7@daegu.ac.kr; 4 Department of Environmental Health Science, New York Medical College, Valhalla, NY 10595, USA; HongDuck_Kim@nymc.edu; 5 Animal Genetic Resources Station, National Institute of Animal Science, RDA, Namwon 590-832, Korea; sungwoo@korea.kr

**Keywords:** annual wormwood leaf, 3T3-L1 cells, adipogenesis, high-fat diet, PPARγ, Akt

## Abstract

Annual wormwood (AW) (*Artemisia annua* L.) has anti-malarial, anti-bacterial, anti-oxidant, anti-tumour, and anti-inflammatory activities. In the present study, we evaluated the effects of annual wormwood leaves (AWL) on adipocyte differentiation in 3T3-L1 cells and high-fat diet (HFD)-induced obese rats. 3T3-L1 adipocytes and HFD-induced obese rats were treated with AWL, and its effect on gene expression was analyzed using RT-PCR and Western blotting experiments. Treatment with AWL effectively prevented triglyceride accumulation during adipogenesis in a dose-dependent manner. Consistently, AWL suppressed the differentiation of 3T3-L1 preadipocytes into adipocytes through the downregulation of dexamethasone, 3-isobutyl-1- methylxanthine, and insulin (DMI)-induced serine/threonine kinase protein kinase B (PKB/Akt) activation and the expression of adipogenic genes, including the CCAAT/enhancer binding protein-α (C/EBPα) and peroximal proliferator-activated receptor-γ (PPARγ). Moreover, the expression of adipocyte fatty acid-binding protein 4 (aP2), which is a known PPARγ-target gene, was downregulated by AWL treatment. Oral administration of AWL extracts significantly decreased the body weight gain, adipose tissue mass, adipocyte cell size, serum triglyceride (TG), and total cholesterol (TC) levels in HFD-induced obese rats. These results provide novel insight into the molecular mechanisms underlying the anti-obesity effects of AWL that are mediated by the downregulation of the expression of major adipogenic transcription factors, C/EBPα and PPARγ and Akt signalling.

## 1. Introduction

Obesity is a condition in which a person has an abnormally high and unhealthy proportion of body fat. Obesity is a major risk factor for many metabolic disorders, including hyperlipidemia, diabetes mellitus, atherosclerosis, hypertension, and cardiovascular disease [1]. Physiologically, obesity is associated with increased levels of adipocytes and an increase in adipocyte volume. Although accumulated intracellular triglycerides can be broken down by exercise or diet, obesity caused by increased fat-cell size and the number is difficult to treat, as the fat cells must be destroyed or removed. 

Adipogenesis is a multi-step process involving a cascade of transcription factors and adipocyte-specific gene expression leading to adipocyte development. Lipid accumulation reflects the process of adipogenesis, which is regulated by genetic and growth factors [2,3]. Adipogenesis is a differentiation process by which preadipocyte cells undergo terminal differentiation to mature adipocytes. CCAAT/enhancer binding protein-δ (C/EBPδ) and CCAAT/enhancer binding protein-β (C/EBPβ) are rapidly and transiently expressed after the hormonal induction of differentiation [4,5]. These genes act synergistically to promote the expression of CCAAT/enhancer binding protein-α (C/EPBα) and peroximal proliferator-activated receptor-γ (PPARγ), which are the master adipogenic transcription factors [6,7]. After differentiation, adipocytes regulate lipid metabolism through lipogenic proteins such as fatty acid synthase (FAS) and aP2 [7]. 

Insulin and Akt signalling modulates adipose tissue growth and adipogenesis [8]. Insulin stimulates glucose and free fatty acid uptake, inhibits lipolysis, and stimulates de novo fatty acid synthesis in adipocytes. The Ser/Thr kinase Akt plays an essential role in adipocyte differentiation. Mouse embryonic fibroblasts (MEFs) lacking Akt display an inability to differentiate into adipocytes [9], and an RNAi-mediated decrease in Akt was found to block the differentiation of 3T3-L1 cells [10]. Glycogen synthase kinase-3β (GSK-3β), which controls glycogen and protein synthesis among many other cellular processes, was one of the first described physiological targets of Akt [11]. 

Recently, the medical use of natural plant products could provide more effective and less expensive medications for people than ever before. *Artemisia annua* L., also known as annual wormwood (AW), is a common type of wormwood that belongs to the family Asteraceae. Annual wormwood leaves (AWL) have been used for many centuries in traditional medicine in Asia in the treatment of febrile diseases and malaria. Among major components such as monoterpenes, camphor, and Artemisia ketone in *Artemisia annua* L., artemisinin, which has a critical role in anti-oxidant and anti-inflammatory with formula C_15_H_22_O_15_ and characterized by structural features, contains a peroxide bridge (C-O-O-C) [12]. As an important biological functional, artemisinin is known to have anti-bacterial, anti-fungal, anti-leishmanial, anti-oxidant, anti-tumour, and anti-inflammatory activities [13,14,15]. In particular, artemisinic acid isolated from AW inhibited adipogenic differentiation of human adipose tissue-derived mesenchymal stem cells [16]. 

Annual wormwood is associated with many health benefits. However, it remains unknown how AWL promotes an anti-obesity effect in 3T3-L1 adipocytes and high fat diet (HFD)-induced obese rats. In the present study, the effect of AWL extracts on adipocyte differentiation in 3T3-L1 cells were investigated by measuring the accumulation of intracellular droplets of triglyceride as well as the expression levels of several adipogenesis-related genes. Moreover, in order to understand the specific mechanisms of these effects, we examined whether Akt and GSK3β activation is critical for the anti-adipogenic functions of AWL. We further evaluate anti-obesity effects of AWL in obese rats fed high-fat diets (HFDs).

## 2. Materials and Methods

### 2.1. Preparation of Annual Wormwood Leaf (AWL) Extracts

Fresh leaves of annual wormwood (AW) were collected immediately after harvesting in May 2016 at Jinju, Gyeongnam (Animal Bio-Resources Bank, Gyeongnam, Korea). Annual wormwood leaves (AWL) were authenticated by Professor T. H. Kim in the Department of Food Science and Biotechnology, Daegu University, Korea. Fresh samples of annual wormwood leaves were prepared by alcohol extraction. The leaves were chopped after washing with running water, dried in oven at 40 °C for 2 days followed by grinding to a powder. The AWL (30 g) powder was then suspended in an 80% (*v*/*v*) ethanol solution using a mixer, followed by extraction of the samples for 3 days with vigorous shaking at room temperature and filtering through Whatman No. 1 filter paper. The ethanolic extracts of AWL were concentrated using rotary-vacuum evaporation at 50 °C and then freeze-dried.

### 2.2. Cell Culture

Mouse 3T3-L1 preadipocytes were purchased from the Korean Cell Line Bank (Seoul, Korea) and cultured as described elsewhere [17]. In brief, cells were cultured in Dulbecco’s Modified Eagle High-glucose Medium (DMEM) supplemented with 10% calf serum at 37 °C in a humidified atmosphere of 5% CO_2_. At 1 day postconfluence (designated “day 0”), cell differentiation was induced with a mixture (DMI) of 0.5 mM 3-isobutyl-1-methylxanthine, 100 μM indomethacin, 0.25 μM dexamethasone and 167 nM insulin in DMEM containing 10% FBS. The 3-isobutyl-1-methylxanthine (MIX), dexamethasone (DEX), indomethacin, and Oil Red O were obtained from Sigma-Aldrich (St. Louis, MO, USA). The medium was changed every 2 days. AWLs were added to the culture medium of the adipocytes on day 0. The cells were treated with 0, 25, or 100 μg/mL of AW extracts every day. After treatment with AWL for 4 and 7 days, the 3T3-L1 adipocytes were lysed for Western blot analysis. To analyse cell viability, the cytotoxicity of the AWL was evaluated using 3-(4, 5-demethylthiazol-2-yl)-2, 5-diphenyltetrazolium bromide (MTT). 

### 2.3. Oil Red O Staining

The cellular lipid content was assessed by Oil Red O staining (Sigma, St. Louis, MO, USA). Cells were treated either with AWL extracts (25 μg/mL or 100 μg/mL) or vehicles in the differentiation medium for days 0–7 of adipogenesis. On days 4 or 7, cells were stained with Oil Red O. For Oil Red O staining, cells were washed gently with phosphate-buffered saline (PBS), and stained with filtered Oil Red O solution (60% isopropanol and 40% water) for 30 min. After staining the lipid droplets red, the Oil Red O staining solution was removed and the plates were rinsed with water and dried. After 3 washes with PBS, cells were photographed with a 12-megapixel digital camera (Canon, Tokyo, Japan). 

### 2.4. Measurement of Triglyceride Content

Cellular triglyceride content was measured using a commercial Triglyceride Assay Kit (Sigma-Aldrich, St Louis, MO, USA) according to the manufacturer’s instructions. Adipocytes differentiated for 4 or 7 days were treated with the AWL at concentrations of 0, 25 and 100 μg/mL in 6-well plates. To analyse the content of cellular triglycerides, cells were washed with PBS and then scraped into 200 μL PBS and homogenized by sonication for 1 min. The lysates were assayed for total triglycerides using the assay kits. 

### 2.5. RT-PCR

RNA was isolated from 3T3-L1 adipocytes or epididymal adipocyte tissue using the RNeasy plus Mini Kit (Qiagen, Valencia, CA, USA) according to the manufacturer’s protocol. Two micrograms of total RNA was used for first-strand cDNA synthesis with oligo (deoxythymidine) primers and Superscript II reverse transcriptase (Invitrogen, Carlsbad, CA, USA). The target cDNA was amplified using the following primers: C/EBPβ, 5′-GACTACGCAACACACGTGTAACT-3′ and 5′-CAAAACCAAAAACATCAACAACCC-3′; PPARγ, 5′-TTTTCAAGGGTGCCAGTTTC-3′ and 5′-AATCCTTGGCCCTCTGAGAT-3′; C/EBPα, 5′-TTACAACAGGCCAGGTTTCC-3′ and 5′-GGCTGGCGACATACAGATCA-3′; aP2, 5′-TGATGCCTTTGTGGGAACCT-3′ and 5′-GCAAAGCCCACTCCCACTT-3′; ACC, 5′-GAATCTCCTGGTGACAATGCTTATT-3′ and 5′-GGTCTTGCTGAGTTGGGTTAGC-3′; FAS, 5′-TGTGAGTGGTTCAGAGGCAT-3′ and 5′-TTCTGTAGTGCCAGCAAGCR-3′; β-actin (control), 5′-GACAACGGCTCCGGCATGTGCAAAG-3′ and 5′-TTCACGGTTGGCCTTAGGGTTCAG-3′. The amplification cycles included denaturation at 95 °C for 50 s, annealing at 55 °C for 1 min and elongation at 72 °C for 50 s. After 30 cycles, the PCR products were separated by electrophoresis on a 1.5% agarose gel for 30 min at 100 V. The gene mRNA levels were normalized using β-actin. The gels were stained with 1 mg/mL ethidium bromide and visualized with UV light using Bio-Rad Gel Doc image analysis software (Bio-Rad Laboratories Inc., Hercules, CA, USA). 

### 2.6. Western Blot Analysis

Western blotting was performed according to standard procedures. Briefly, cells were lysed in lysis buffer containing 50 mM Tris-HCl (pH 8.0), 0.4% Nonidet P-40, 120 mM NaCl, 1.5 mM MgCl_2_, 0.1% sodium dodecyl sulfate (SDS), 2 mM phenylmethylsulfonyl fluoride, 80 μg/mL leupeptin, 3 mM NaF and 1 mM Dithiothreitol (DTT). Cell lysates (50 μg protein) were separated by 10% SDS-polyacrylamide gel electrophoresis, transferred onto a polyvinylidene fluoride membrane (Amersham Pharmacia, Little Chalfont, England, UK), blocked with 5% skim milk and hybridized with primary antibodies. PPARγ, C/EBPβ, C/EBPα, aP2, Akt, and GSK3β antibody were from Cell Signaling (Danvers, MA, USA) and the monoclonal β-actin antibody was from Chemicon (Temecula, California, USA). Horseradish peroxidase (HRP)-labelled mouse anti-rabbit IgG were from Jackson ImmunoResearch (West Grove, PA, USA). The Chemiluminescence Kit was from Pierce (Rockford, IL, USA). After incubation with horseradish-peroxidase-conjugated secondary antibody at room temperature, immunoreactive proteins were detected using a chemiluminescent ECL Assay Kit (Amersham Pharmacia, Little Chalfont, England, UK) according to the manufacturer’s instructions.

### 2.7. Animals and Diets

Four-week-old Sprague–Dawley male rats were purchased from Central Lab Animal Inc. (Seoul, Korea). All animal experiments were performed following the ethical guidelines set out by the Gyeongsang National University’s institutional animal care and with the approval of the Animal Care and Use committee of Gyeongsang National University (Approval Number: GNU-160912-R0032). The experiments began after acclimating the animals for 7 days under constant conditions of temperature (22 °C), humidity (55%), and light (12 h cycle dark/light) in polycarbonate cages. The animals were randomly divided into three groups (*n* = 10) and fed the normal or experimental diets for 5 weeks as follows: (1) a normal diet group (ND, *n* = 10); (2) a high-fat diet group (HFD, *n* = 10); (3) a AWL group (HFD + AWL 150 mg/kg BW, *n* = 10). Rats in the ND group were fed a normal diet (#55VXT0038, Samyang Co., Seoul, Korea). Feeding rats a high-fat diet produced obese rats, and rats in the HFD groups were fed an HFD based on a commercial diet (rodent diet with 60% kcal fat, Research Diet, Seoul, Korea). The animals were allowed free access to food and water for five weeks. Food intake was measured daily, and the rats were weighed twice per week. At the end of the experiment period, rats were sacrificed after 12 h of fasting. 

### 2.8. Biochemical Analysis 

Whole blood samples were centrifuged in a tube containing heparin as anti-coagulant, and isolated serum was used for analysis of triglyceride (TG), total-cholesterol (TC), and high-density lipoprotein-cholesterol (HDL-C). After centrifugation, the organic layer was removed and dried. The resulting pellet was dissolved in phosphate-buffered saline containing 1% Triton X-100, and the triglyceride content was determined using a commercially available Enzymatic Reagent Kit (Asan phams, Co., Hwaseon-si, Korea). The concentrations of total-cholesterol (TC) and high-density lipoprotein-cholesterol (HDL-C) were assayed enzymatically using the commercial kits (Asan phams, Co., Korea).

### 2.9. Histological Analysis

Epididymal fat tissues were removed and fixed in 10% neutral-buffered formalin. The fat pads were subsequently embedded in paraffin, sectioned into 5 μm sections (Leica, Wetzlar, Germany), and stained with haematoxylin-eosin for microscopic assessment (Olympus, Tokyo, Japan). Three different cross-sectional areas and their corresponding cell populations were assessed using an image analysis program (Image-Pro Plus Version 6.0, Rockville, MD, USA).

### 2.10. Statistical Analysis

Each experiment was performed at least three times. The data are expressed as the mean ± SD. One-way ANOVA and the Duncan’s multiple tests were used to determine the significant differences between the treatment groups. A *p*-value < 0.05 was considered statistically significant. 

## 3. Results

### 3.1. Inhibition of Lipid Accumulation by AWL in 3T3-L1 Adipocytes 

**Figure 1:**
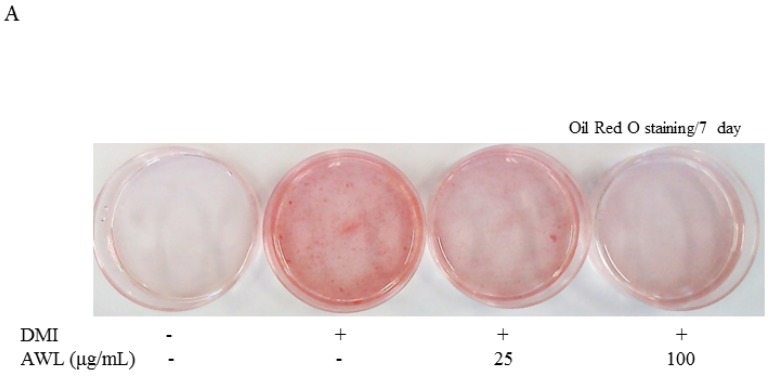

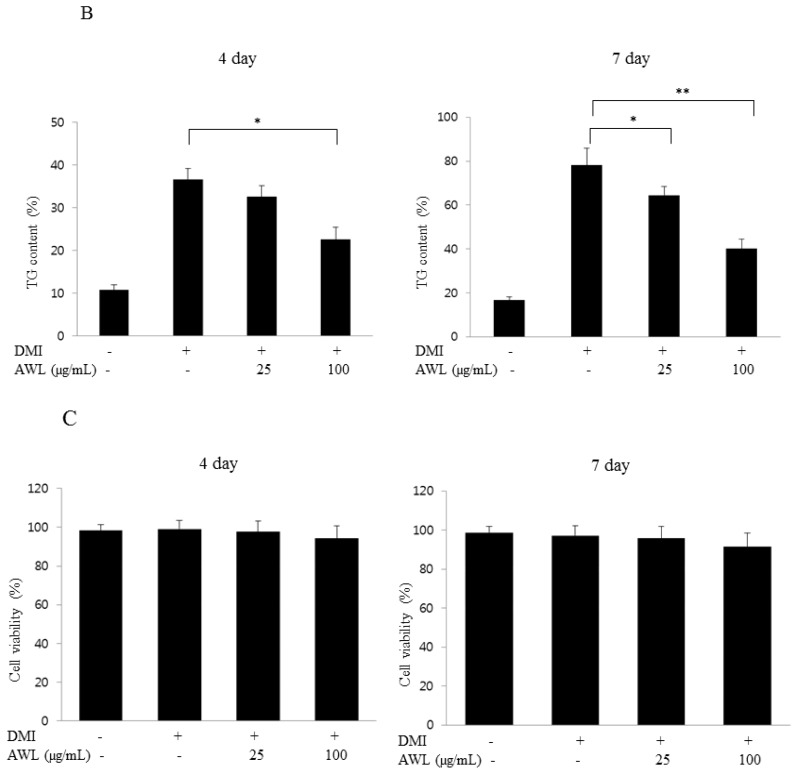
Effects of AWL on lipid accumulation and adipocyte differentiation in 3T3-L1 cells. (**A**). 3T3-L1 cells were induced to differentiate with DMI and AWL in increasing concentrations (0, 25, and 100 μg/mL) for seven days. The cellular lipid content was assessed by Oil Red O staining. DMI: 0.5 mM 3-IBMX, 100 μM indomethacin, 0.25 μM dexamethasone and 167 nM insulin. AWL: annual wormwood leaves extracts. (**B**) AWL inhibited TG accumulation in 3T3-L1 adipocytes. Three independent experiments were used to represent the error bars. * *p* < 0.05, ** *p* < 0.01. (**C**) cytotoxicity of AWL in 3T3-L1 cells. Viability of the AWL-treated cells was determined by the MTT assay. The values are presented as the means ± SD. The data shown are representative of at least three independent experiments.

Two-days post confluence, 3T3-L1 cells were treated with indicated concentrations of AWL and then stimulated with DMI mixture for 7 days. 3T3-L1 cells were stained with the triglyceride-specific Oil Red O 4 or 7 days after the induction of adipocyte differentiation. The quantitation of Oil Red O staining indicated that treatment with the 25 and 100 μg/mL concentration of AWL markedly attenuated adipocyte differentiation, and treatment with AWL (25, 100 μg/mL) induced a dose-dependent decrease in lipid levels, as assessed by an strong decrease in 100 μg/mL AWL-treated cells (Figure 1A). In particular, by day 7, differentiated 3T3-L1 cells revealed a 4–5-fold greater constitutive level of triglyceride content when compared to undifferentiated cells. However, intracellular triglyceride accumulation was decreased by 43% in the 100 μg/mL AWL treated 3T3-L1 adipocyte compared with fully differentiated adipocytes (Figure 1B). A cell viability assay was performed to examine the possibility that the AWL effect was simply a consequence of cytotoxicity. At a concentration of 100 μg/mL, AWL had no significant activity on cell viability compared to controls and did not cause cytotoxicity in the 3T3-L1 cells after 4 or 7 days of incubation (Figure 1C). Together, these results demonstrate that AWL exerted an anti-adipogenic effect on 3T3-L1 adipocytes. 

### 3.2. Effects of AWL on the mRNA and Protein Levels of Genes Involved in Adipogenesis and Lipogenesis in 3T3-L1 Cells

**Figure 2:**
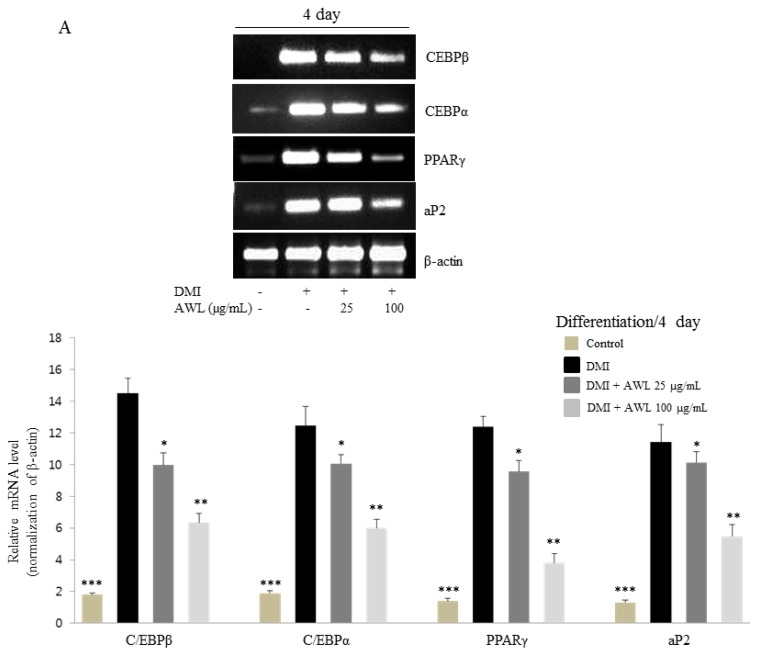

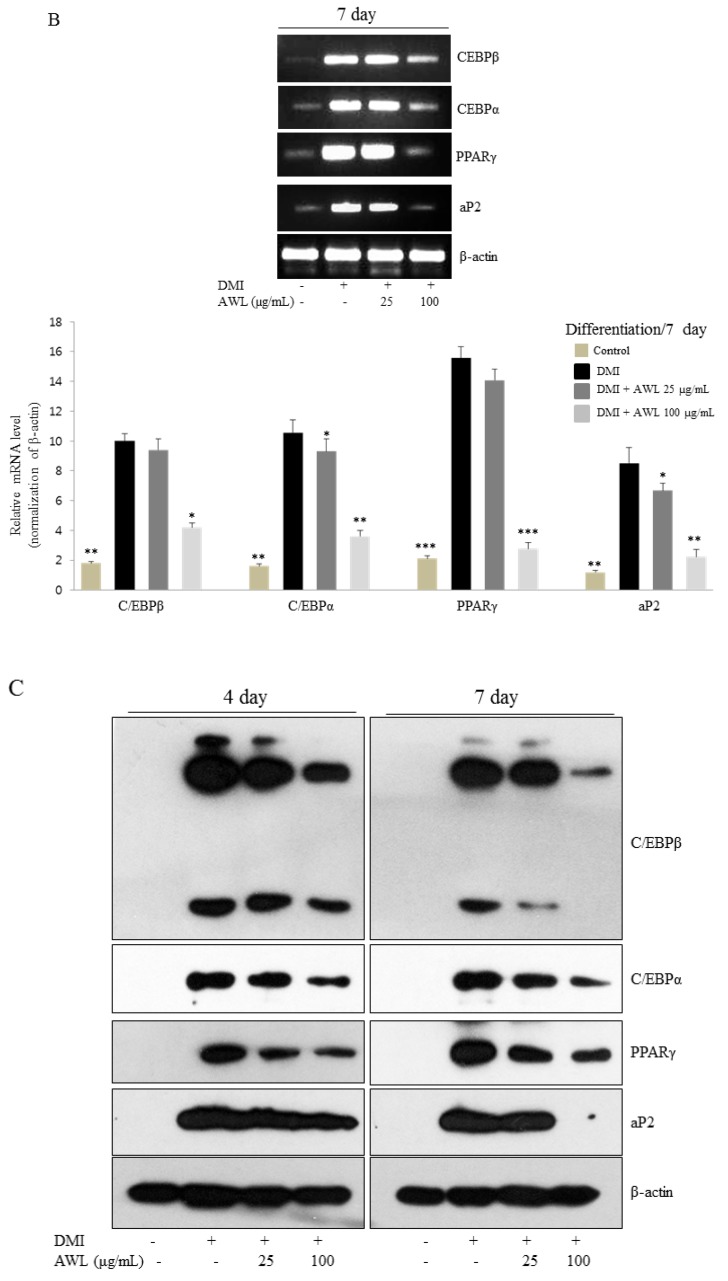
Effects of AWL on adipogenic factors in 3T3-L1 adipocytes. (**A**) AWL decreased the expression of adipogenic factors in 3T3-L1 adipocytes at day 4 by RT-PCR. Similar results were obtained by three independent experiments; (**B**) AWL inhibited the expression of adipogenic factors in 3T3-L1 adipocytes at day 7 by RT-PCR. The figure is representative of three independent experiments with identical results. Optical density analysis was performed to quantify the levels of mRNA expression with β-actin as loading control. * *p* < 0.05, ** *p* < 0.01, and *** *p* < 0.001 compared with DMI group. (**C**) AWL inhibited the expression of genes related to adipogenesis and lipogenesis in 3T3-L1 adipocytes at days 4 and 7 as determined by a Western blot assay.

To examine the effect of AWL on adipogenic genes, we investigated the expression levels of C/EBPβ, C/EBPα, PPARγ, and aP2 in 3T3-L1 cells cultured in the presence or absence of AWL (25 or 100 μg/mL) with adipocyte differentiation for four or seven days. We found that the expression levels of C/EBPβ, C/EBPα, PPARγ, and aP2 mRNA increased in differentiated 3T3-L1 adipocytes. However, after treatment with AWL (25 or 100 mg/mL) for four or seven days, C/EBPβ, C/EBPα, PPARγ, and aP2 level were significantly decreased during differentiation to adipocytes in 3T3-L1 cells (Figure 2A,B). Next, to examine the protein expression patterns of adipogenic-specific genes during 3T3-L1 differentiation, protein levels of C/EBPβ, C/EBPα, and PPARγ were measured by Western blot analysis. The expression levels of C/EBPβ, C/EBPα, and PPARγ were dose-dependently reduced after AWL treatment for four or seven days (Figure 2C). These results indicated that AWL blocked 3T3-L1 adipocyte differentiation via the downregulation of C/EBPβ, C/EBPα, and PPARγ expression. Moreover, to determine whether the repression of PPARγ and C/EBPα cause the downregulation of their target gene, aP2, we examined the activation of aP2 under the same conditions. Treatment with AWL significantly reduced aP2 expression compared to that of the fully differentiated 3T3-L1 adipocytes (Figure 2C). 

### 3.3. Effect of AWL on Akt and GSK3β Phosphorylation during Adipocyte Differentiation

**Figure 3:**
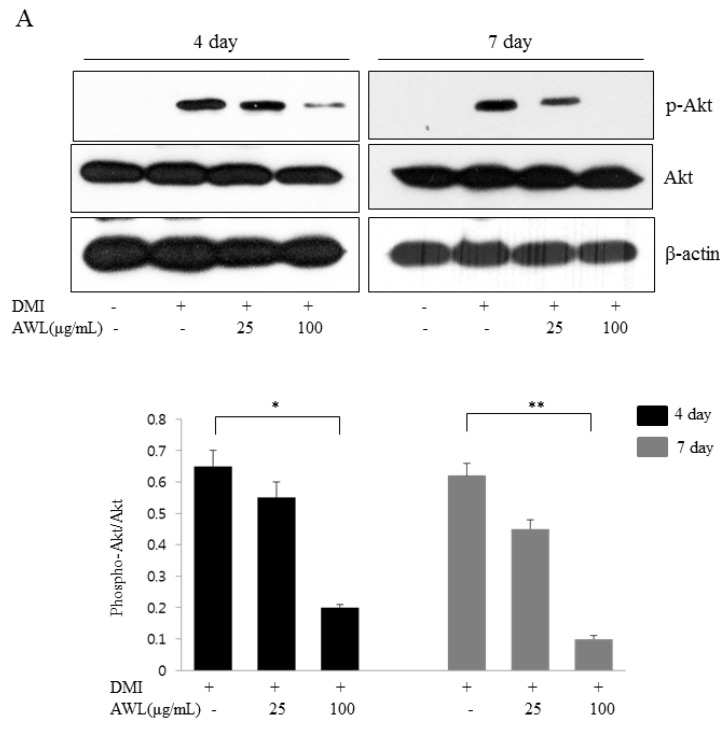

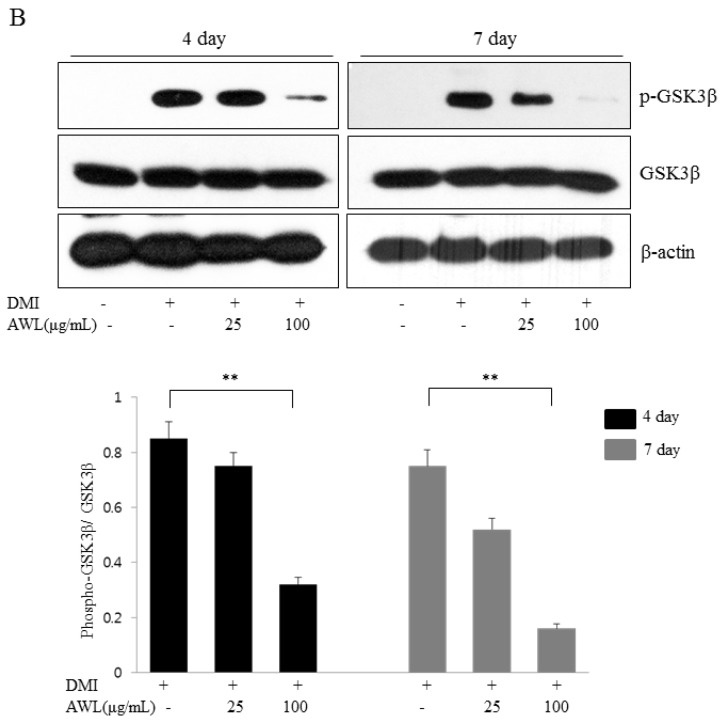
Effects of AWL treatment on insulin-stimulated Akt and GSK3β phosphorylation in 3T3-L1 adipocytes. (**A**) AWL inhibited the Akt signalling pathways in a dose-dependent manner in 3T3-L1 adipocytes. 3T3-L1 cells were differentiated with DMI in the absence or the presence of AWL for 4 or 7 days. The Akt phosphorylation was normalized to the total Akt expression level. * *p* < 0.05, ** *p* < 0.01. (**B**) AWL inhibited the phosphorylation of GSK3β in 3T3-L1 adipocytes. The phosphorylation of GSK3β was normalized to the total GSK3β expression level. The data shown are representative of at least three independent experiments. * *p* < 0.05, ** *p* < 0.01.

The Akt pathway has an important role in controlling adipogenesis. In the present study, we determined whether Akt phosphorylation was involved in AWL-induced adipocyte differentiation blockage. 3T3-L1 cells were induced to differentiate with various concentrations of AWL (0, 25, and 100 μg/mL) in the presence of DMI or DMI mixture alone. We investigated the protein expression of Akt, GSK3β and their phosphorylation forms. In control cells, DMI-stimulated 3T3-L1 adipocytes showed a significant increase in phospho-Akt (Ser473) and phospho-GSK3β (Ser9) (Figure 3A,B). In contrast, AWL treatment strongly inhibited the phosphorylation levels of Akt and GSK3β, while AWL had no inhibitory effect of total Akt and GSK3β expression (Figure 3A,B). These results suggest that AWL inhibited the phosphorylation of Akt, which was associated with the suppressed phosphorylation of its substrate kinase GSK3β. 

### 3.4. AWL Reduced Body Weight Gain, Adipose Tissue Mass and Adipocyte Size in HFD-Induced Obese Rats

**Figure 4:**
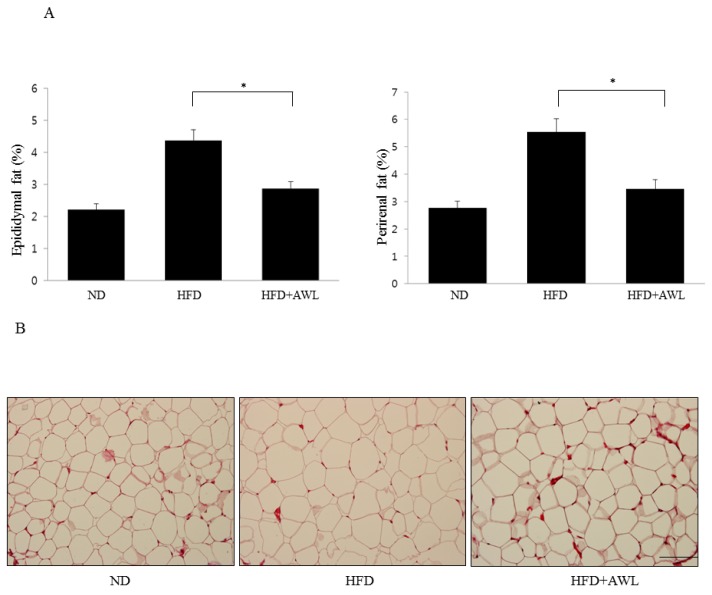
AWL ameliorates adiposity in HFD-induced obese rats. Rats were fed a normal diet or high-fat diet for five weeks in the presence (150 mg/kg/day) or absence of AWL (*n* = 10). (**A**) the weight of perirenal and epididymal fat was significantly reduced by AWL treatment. * *p* < 0.05; (**B**) histological analysis of epididymal adipose tissue after staining with Haematoxylin and Eosin (H&E) followed by microscopy analysis. Scale bar is 100 μm.

We examined the anti-obesity effects of AWL in HFD-induced obese rats. Body weight gain in the HFD-fed group was higher than the body weight gain in the ND control group. In particular, treatment with 150 mg/kg of AWL to HFD-fed rats for five weeks by oral administration significantly reduced body weight gain (Table 1). After five weeks on the HFD, the body weights of the HFD plus AWL group were 17% lower than that of the HFD group. Food intake was not significantly different among all groups. These results demonstrated that AWL reduced body weight gain without affecting food intake. To examine whether decreased body weight gain in the AWL-fed group compared to the HFD group was associated with a decrease in fat accumulation, the weights of epididymal and perirenal adipose tissues and the sizes of epididymal adipocytes were measured. We observed that supplementation with 150 mg/kg of AWL markedly reduced epididymal and perirenal fat mass (40.2% and 32.3%) compared to those of the HFD group (Figure 4A), demonstrating that the reduction in body weight gain was primarily due to a decrease of fat accumulation in adipocytes. In obese rats, the adipocyte sizes were validated by histological examination of the epididymal fat tissue. These results demonstrated that the sizes of the adipocytes in the AW group were significantly reduced compared that in the HFD group (Figure 4B). 

### 3.5. Effect of AWL on Serum Triglyceride and Cholesterol Content in HFD-Induced Obese Rats

**Figure 5:**
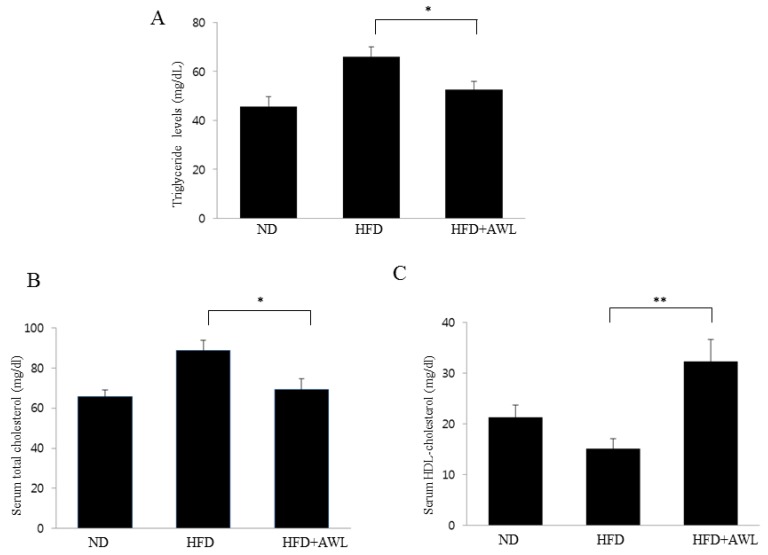
Effects of AWL treatment on lipid content in HFD-induced obese rats. (**A**–**C**) significant decreases were observed in serum TG and TC levels of the AWL-treated group compared to the HFD alone group. HDL-C levels were significantly higher in the AWL-treated group compared to that in the HFD alone group. Statistical significance between the HFD alone group and HFD plus AWL group was calculated using Duncan’s multiple range test. * *p* < 0.05, ** *p* < 0.01.

Similar to the results for body weight gain and fat mass, serum TG and TC levels were significantly higher and HDL-C was dramatically lower in the HFD group compared to the ND group. The treatment of AWL significantly reduced the serum TG and TC levels in HFD plus AWL group compared to the HFD alone group, while serum HDL-C levels were significantly increased in the HFD plus AWL group compared to the HFD group (Figure 5). 

### 3.6. Effects of AWL on the mRNA Expression in White Adipose Tissue

**Figure 6:**
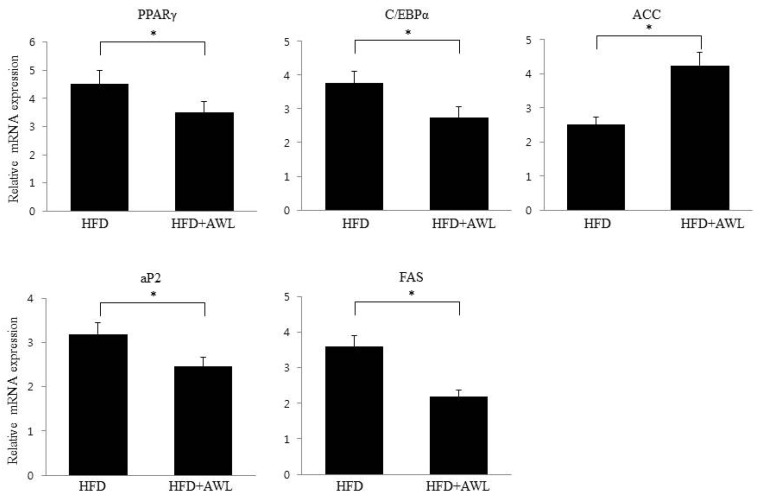
Effects of AWL on mRNA expression in epididymal adipose tissues. AWL decreased the expression of adipogenic factors in adipocytes. PPARγ, peroximal proliferator-activated receptor-γ; C/EPBα, CCAAT/enhancer binding protein-α; ACC, acetyl-CoA carboxylase; aP2, adipocyte fatty acid-binding protein 4; FAS, fatty acid synthase. The values are presented as the means ± SD. The data shown are representative of at least three independent experiments. β-actin expression in each sample was used as an internal control to normalize expression. * *p* < 0.05.

In order to further investigate the anti-obesity effects of AWL, we compared the expression levels of adipogenesis-related genes in adipocyte tissue in HFD-treated and HFD plus AWL-treated rats. Gene expression of PPARγ and C/EBPα, major regulators related to lipid synthesis and TG accumulation in adipocytes, were significantly suppressed in the HFD plus AWL group compared to HFD alone group (Figure 6). In addition, expression of Acetyl-CoA carboxylase (ACC), which is a regulator of fatty acid oxidation, was markedly increased by AWL (Figure 6). We further investigated whether the AWL-induced reduction of PPARγ and C/EBPα regulated the expression of their target gene, such as FAS and aP2. As expected, AWL suppressed the mRNA expression levels of aP2 and FAS in adipose tissue of HFD–induced obese rats. Taken together, these findings indicated that AWL could downregulate the expression of the genes involved adipogenesis and lipid metabolism in adipocytes. 

## 4. Discussion

Obesity is a risk factor for multifaceted metabolic syndromes including Hyperlipidaemia, Type II Diabetes, and Cardiovascular Disease (CVD) [1,2]. To date, considerable amount of advancement on target discovery using metabolomics platform with various analytical technologies has been made in the field of metabolic disorder; this includes obesity. Compelling evidence supports that natural substances and herb compounds have focused on prevention and treatment of obesity to achieve a healthy lifestyle. Ongoing high-throughput drug screening in biomedicine leads to a major bottleneck of synthetic chemistry strategies-driven several medications. This reflects tens of thousands of anti-obesity compounds that have been withdrawn due to their serious side effects (biological activity vs. toxicity). Therefore, trends of recent studies have focused on screening natural products to explore functional core component resulting in body weight loss and reduction of levels of fat that generally have minimal adverse effects [18]. In similar context, we have investigated the underlying molecular mechanism of *Artemisia annua* L. (AWL), a potential anti-obesity effect, which could be associated with changes in a molecular level including transcriptional factors and regulatory pathway in adipocyte differentiation and lipogenesis using 3T3-L1 adipocytes as cellular models and HFD-induced obese rats in animal models. 

Continuous efforts to elucidate molecular network of obesity accumulated evidence that a key feature of secondary metabolites from *Artemisia annua* L. (AWL) possesses a number of biological activities [19,20]. For example, it can exhibit anti-proliferative effects or induce apoptosis in various tumour cell lines in vitro and in vivo [21,22]. Additionally, *Artemisia annua* L. (AWL) has demonstrated significant anti-oxidant, anti-inflammatory, and anti-microbial properties [12]. Recent studies also reported that artemisinic acid isolated from *Artemisia annua* L. (AWL) inhibited adipogenic differentiation of human adipose tissue-derived mesenchymal stem cells [16]. Although numerous studies have demonstrated that AWL has a potential role as an anti-cancer therapy, there is limited information available about the anti-obesity effects of AWL.

As a key biological target, adipose tissue plays an important role as a storage depot for excess energy and regulates body energy homeostasis. Adipocyte hyperplasia and hypertrophy, as a result of inadequate adipogenesis in fat tissues, lead to obesity. Adipocyte differentiation from preadipocyte into mature adipocytes is a highly controlled process that involves changes in gene expression and the enlargement of intracellular lipid droplets [23]. In the present study, we confirm inhibition of lipid accumulation by measuring intracellular triglyceride level staining with Oil Red O in 3T3-L1 adipocytes. This could lead to a significant reduction in adipogenesis in a dose-dependent manner of AWL without cell cytotoxicity. Furthermore, our in vivo study showed marked interventions in which AWL strongly reduced body weight gain, adipose mass, and adipocyte hypertrophy. Therefore, our findings suggest that AWL exerted anti-obesity effects in adipocytes and in HFD-induced obese rats. 

Several transcription factors regulate adipocyte differentiation, in particular, C/EBPs and PPARγ, which synergistically activate the expression of adipocyte-specific genes to transform preadipocyte into mature adipocytes [24]. PPARγ is a ligand-activated transcription factor that regulates adipogenesis during the early to terminal phase of differentiation. C/EBPα is expressed in the mid to late stages of adipogenesis and stimulates the differentiation of preadipocytes in cooperation with PPARγ [6]. Moreover, PPARγ-deficient cells fail to differentiate into adipocytes, and overexpression of PPARγ, while C/EBPα accelerates adipogenesis [25]. In this study, our findings unfold the molecular regulation of obesity by which AWL effectively inhibited the expression of C/EBPβ, C/EBPα, and PPARγ at the mRNA and protein levels. PPARγ and C/EBPα synergistically trans activate downstream adipocyte-specific gene expression, including aP2 enhancer, which is directly associated with lipogenic pathways [26]. These results manifested the molecular link of obesity through the treatment of AWL inhibited the expression of aP2, which facilitate AWL driven suppression of adipogenesis via the downregulation of C/EBPα and PPARγ on molecular dysregulation in disease complications such as obesity.

Obesity causes insulin resistance, which may be associated with health complications such as diabetes, hyperlipidemia, and hypertension. There is a clear premise that the insulin signalling pathway plays a critical role in insulin-induced adipogenesis. Akt is an important signal mediator in the insulin-like growth factor 1 receptor signal cascade, which is involved in the induction of adipocyte differentiation [27]. Ectopic expression of activated Akt induces the differentiation of 3T3-L1 pre-adipocytes into adipocytes [28,29]. Moreover, the overexpression of PPARγ in Akt-deficient mouse embryonic fibroblasts rescued their severe adipogenesis defect [30], which supports the essential role of PPARγ induction downstream of Akt. In the present study, we found that AWL treatment induced a dose-dependent decrease in Akt phosphorylation and subsequently reduced the phosphorylation level of GSK3β. Both observations could be partly involved in the dysfunction of adipogenesis or lipogenesis. Emerging results including us, AWL-induced functional inactivate, such as inhibition of PPARγ and C/EBPα expression, might be influenced to the decreased level of Akt phosphorylation. Therefore, our results provide the first evidence that AWL inhibited insulin-mediated Akt phosphorylation, which, in turn, dramatically reduced adipogenic triglyceride accumulation by downregulating the PI3K/Akt pathway during the differentiation of 3T3-L1 preadipocytes into adipocytes. 

Consistent with the inhibitory effect of AWL on adipocyte lipid accumulation, an in vivo study indicated that AWL prevented obesity in HFD-induced obese rats. The body weight of rats fed an HFD was lowered by 17% following administration of AWL compared to the HFD alone group. The reduced weight gain in AWL-fed rats was accompanied by a reduction in the weights of epididymal and perirenal adipose tissues. We examined the effect of AWL on adipogenesis- and/or obesity-related gene expression in adipose tissue of HFD-induced obese rats. The expressions of PPARγ and C/EBPα in adipose tissue of the AWL group were downregulated; subsequently, the gene expression of aP2 and FSA were decreased by treatment with AWL. Moreover, ACC, a PPAR-regulated fatty acid oxidation gene, was upregulated. Thus, our findings suggest that AWL significantly suppressed the expression of PPARγ and C/EBPα genes, which may regulate adipogenesis-related gene expression, fatty acid synthase, and lipogenesis in adipocytes. We also observed that AWL administration resulted in the improvement of numerous serum metabolic parameters, decreasing serum TG, TC, and increasing HDL-C levels, which are used as an indicator of adipocyte lipolysis. In addition, histological examination showed smaller fat cells in the epididymal fat tissue of the AWL-fed group in comparison to that in the HFD alone group, indicating that the decreased body weight gain was due to the reduced accumulation of fat. According to our observations, both natural and synthetic compounds from AWL shed light on new avenues of anti-obesity progression and/or blunt underlying obesity linked insulin resistance underlying metabolic health complications.

## 5. Conclusions

In summary, our data demonstrated that AWL suppressed 3T3-L1 adipogenic differentiation via the inhibition of Akt phosphorylation and C/EBPs and PPARγ expression. Moreover, AWL attenuated HFD-induced weight gain, fat deposition, and adipose cell size, and alleviated serum TC, TG, and HDL-C levels. These results that were obtained from in vivo and in vitro levels suggest that AWL could significantly enhance pathophysiological symptoms in obesity animal models and thus may be an effective alternative therapeutic agent in preventing obesity and other related metabolic disorders. 

## Figures and Tables

**Table 1:** Effect of AWL on body weight and food intake in rats fed HFDs for five weeks. The effect of AWL on body weight and food intake in rats fed HFD for five weeks. Values are presented as the mean ± S.D. (*n* = 10). ^a,b,c^ Means not sharing common letters are significantly different among the groups at *p* < 0.05.

	ND Group	HFD Group	HFD + AWL Group
Food intake (g/kg)	22.1 ± 0.54	21.05 ± 0.34	20.05 ± 0.44
Initial Body weight (g)	107.3 ± 3.7	107.8 ± 4.3	106.7 ± 3.8
Final body weight (g)	316.5 ± 16.8 ^a^	411.5 ± 24.2 ^c^	342 ± 20.5 ^b^
